# PATIENT AND CAREGIVER PERCEPTIONS OF MOBILITY AFTER DISCHARGE FROM SKILLED NURSING FACILITIES TO HOME

**DOI:** 10.1093/geroni/igac059.083

**Published:** 2022-12-20

**Authors:** Ayomide Bankole, John Preisser, Ying Zang, Mark Toles

**Affiliations:** University of North Carolina-Chapel Hill, Chapel Hill, North Carolina, United States; University of North Carolina- Chapel Hill, Chapel Hill, North Carolina, United States; University of North Carolina at Chapel Hill, Chapel Hill, North Carolina, United States; UNC Chapel Hill, Chapel Hill, North Carolina, United States

## Abstract

Functional outcomes of older adults in post-acute care is poorly understood. The purpose of this study was to describe skilled nursing facility (SNF) patient or caregiver perceptions of patient mobility and factors associated with patient mobility in 30 days after discharge from SNF to home. We conducted a secondary analysis of data from a stepped-wedge cluster randomized trial of the Connect-Home intervention set in 6 SNFs in North-Carolina. The sample included SNF patients (N=249) and their caregivers (N=249). In 30 days after discharge, SNF patients or their caregivers (as proxies) rated patient mobility using the Life-Space Assessment (LSA). LSA total-scores (range= 0-120), with higher scores indicating greater mobility. Linear mixed models, with random effects to adjust for clustering in SNFs, were used to describe associations between LSA total-score and patient, caregiver, and health-system factors. Participants were typically female (63%), White (74%), and average age of 76.3 years. In 30 days after discharge, LSA total-scores ranged from 0-90 with a mean score of 22 (SD=16.01). Most (85%) required caregiver and/or assistive devices to transfer inside and outside of their homes. In the mixed models, lower LSA scores were associated with older age [-0.232(0.108), p< 0.05], lower cognition score [1.182(0.384), p< 0.005], diagnosis of dementia [-6.863(3.057), p< 0.05], lower baseline mobility in the SNF [0.296(0.142), p< 0.05] and longer SNF stay [-0.200(0.068), p< 0.005]. Low LSA scores in this sample suggest the need for caregiver support to continue rehabilitation and promote functional ability at home. Studies are needed to augment transitional care and address these care needs.

